# The Writ of Habeas Corpus Denied to Birkholz—Cannot Escape the Medical Practice Act That Way—Must Try Another Plan

**Published:** 1898-09

**Authors:** 


### The Writ of Habeas Corpus Denied to Birkholz—Cannot Escape the Medical Practice Act That Way—Must Try Another Plan.

---

Early in November, 1897, Zwigtman Van Noppen, an unregistered practitioner of Chicago, was prosecuted by the State Board of Health for practicing medicine without a license and fined $100 and costs. In default of payment he was committed to jail. His attorney sued out a writ of habeas corpus, claiming that the following section of the act regulating the practice of medicine was unconstitutional:

Section 14. Judgment Upon Conviction—Appeal—Upon conviction of either of the offenses mentioned in this act the court shall, as a part of the judgment, order that the defendant be committed to the common jail of the county until the fine and costs are paid, and upon failure to pay the same immediately the defendant shall be committed under said order.

The case was heard by Judge Gibbons, of Cook County, on November 12, and a decision rendered in favor of the defendant, who was released.

Although this decision was not the law it was published far and wide as such by the alleged Medical College in Chicago to which Van Noppen was attached and it was stated that the Medical Practice act could no longer be enforced, hence every one who wished could practice in the State unrestricted. The State Board of Health had no redress in the case, as there is no appeal from a habeas corpus decision.

On June 7, the State Board of Health, through its attorney, John A. Barnes, prosecuted A. W. Birkholz for practicing medicine without a license. Birkholz was fined $100 and costs. His attorney, James Lane Allen, who defended Van Noppen, emboldened by his success in that case, started a habeas corpus proceeding in the Supreme Court, being under the impression that a similar decision would be obtained and that the law requiring all practitioners to secure a license would be rendered inoperative. The petition was denied by Judge Phillips, who decided that a writ of habeas corpus would not lie to relieve defendant from the operation of the medical practice act, and that the only remedy would be an appeal from the decision of the court in which Birkholz was convicted.

This decision, which is of great importance, will prevent habeas corpus proceedings being taken in the future. The law has been pronounced constitutional by several eminent lawyers, and the attorney and members of the State Board of Health have very little doubt that should the case ever come before the Supreme Court a similar decision will be rendered.

Attorney Barnes has filed suit in fifty cases against violators of this act in Chicago, and will vigorously prosecute all offenders who are reported to the board.—*Springfield* (*Ill.*), *Monitor*, June 10, 1898.

